# Supplementary material for: Targetable NOTCH1 rearrangements in reninoma
Source: Nat Commun. 2023 Sep 25;14:5826. doi: 10.1038/s41467-023-41118-8 (PMC10519988; doi:10.1038/s41467-023-41118-8)
Supplement: Supplementary file 1 — Supplementary Information [file 41467_2023_41118_MOESM1_ESM.pdf]

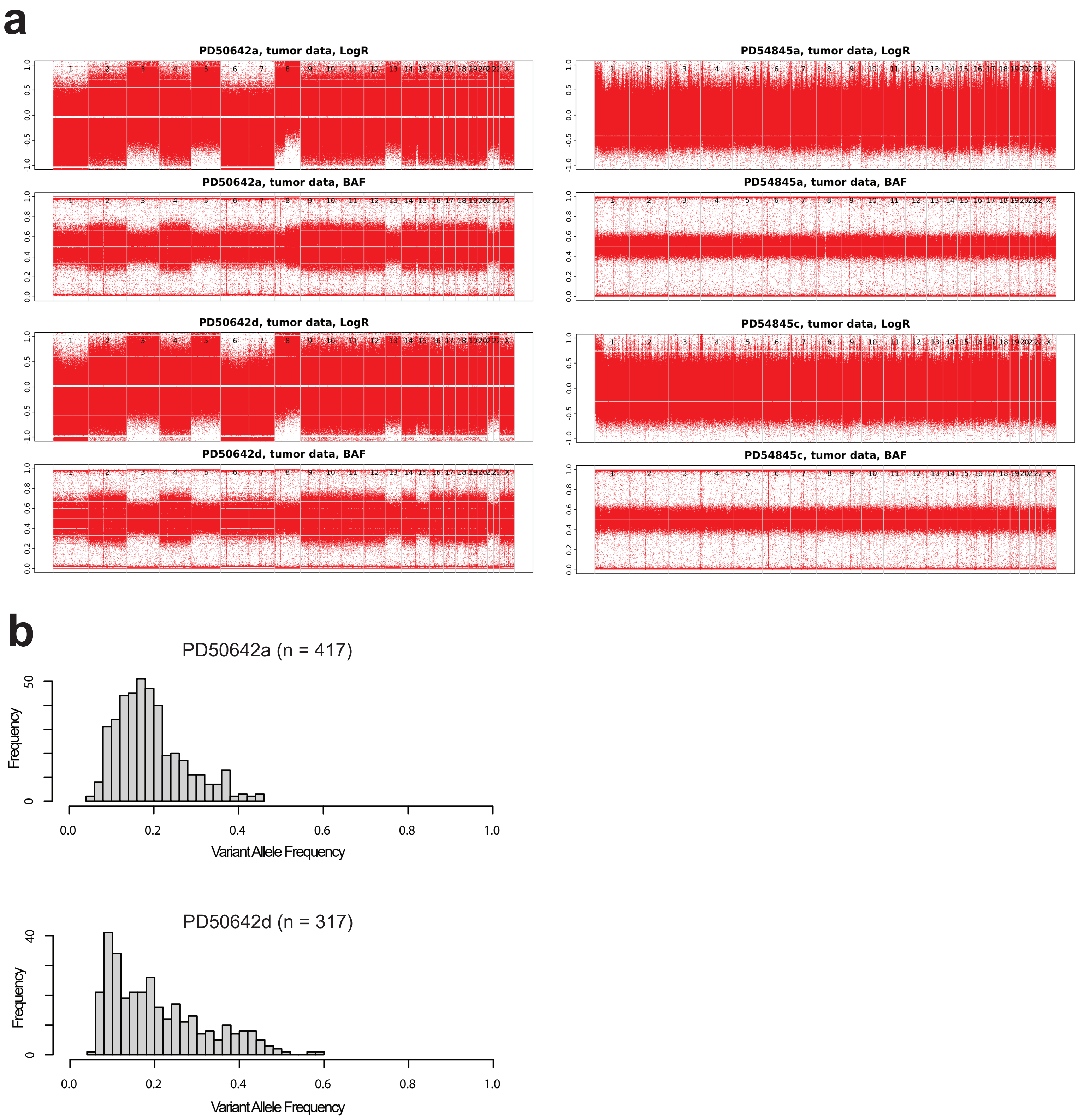

Supplementary Figure 1 - Raw copy number profiles and Variant allele frequency distributions
a, Log R Ratio (LRR) and B Allele frequency (BAF) plots for case 1 and case 2
b, Variant allele frequency (VAF) distribution of substitutions for case 1
Source data are provided as a Source Data file

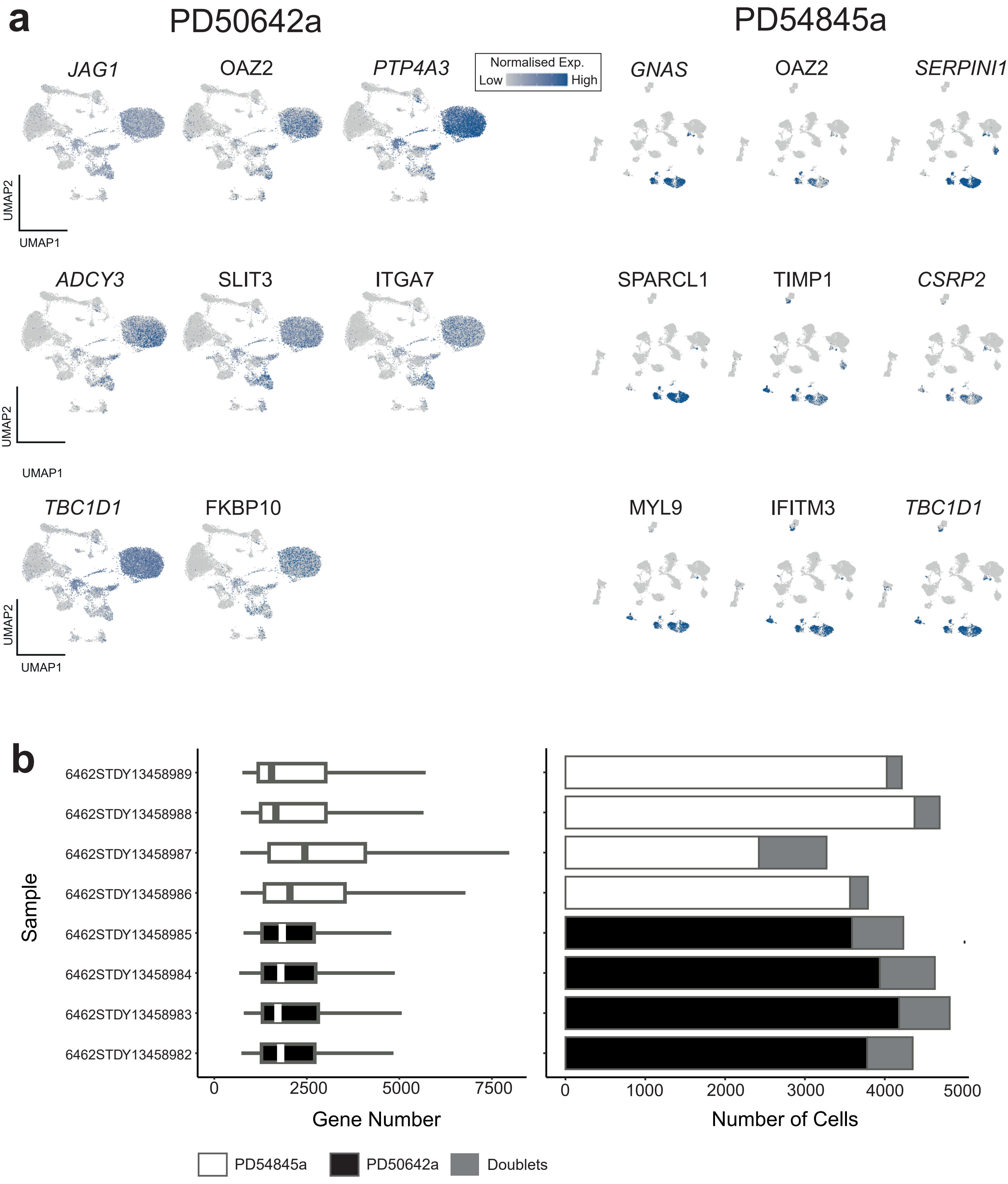

Supplementary Figure 2 - Single nuclear sequencing quality control and expression of classical reninoma markers in tumour cells

a, Uniform Manifold Approximation and projection (UMAP) plots showing normalised expression of reninoma marker genes in tumour cells **b**, Boxplot and barchart showing the post-QC gene number (left panel) and raw cell number with doublet count (right panel) for each sequenced 10x channel, coloured by tumour sample. 6462STDY13458989, n=4026; 6462STDY13458988, n=4373; 6462STDY13458987 ,n=2423; 6462STDY13458986, n=3565; 6462STDY13458985, n=3590; 6462STDY13458984, n=3938; 6462STDY13458983, n=4180; 6462STDY13458982, n=3780. The box contains the 25th to 75th percentiles of the data, with the central line denoting the median value. The upper whisker extends from the median to the largest value, no further than 1.5 * inter-quartile range (IQR). The lower whisker extends from the median to the smallest value, at most 1.5 * IQR. Source data are provided as a Source Data file.

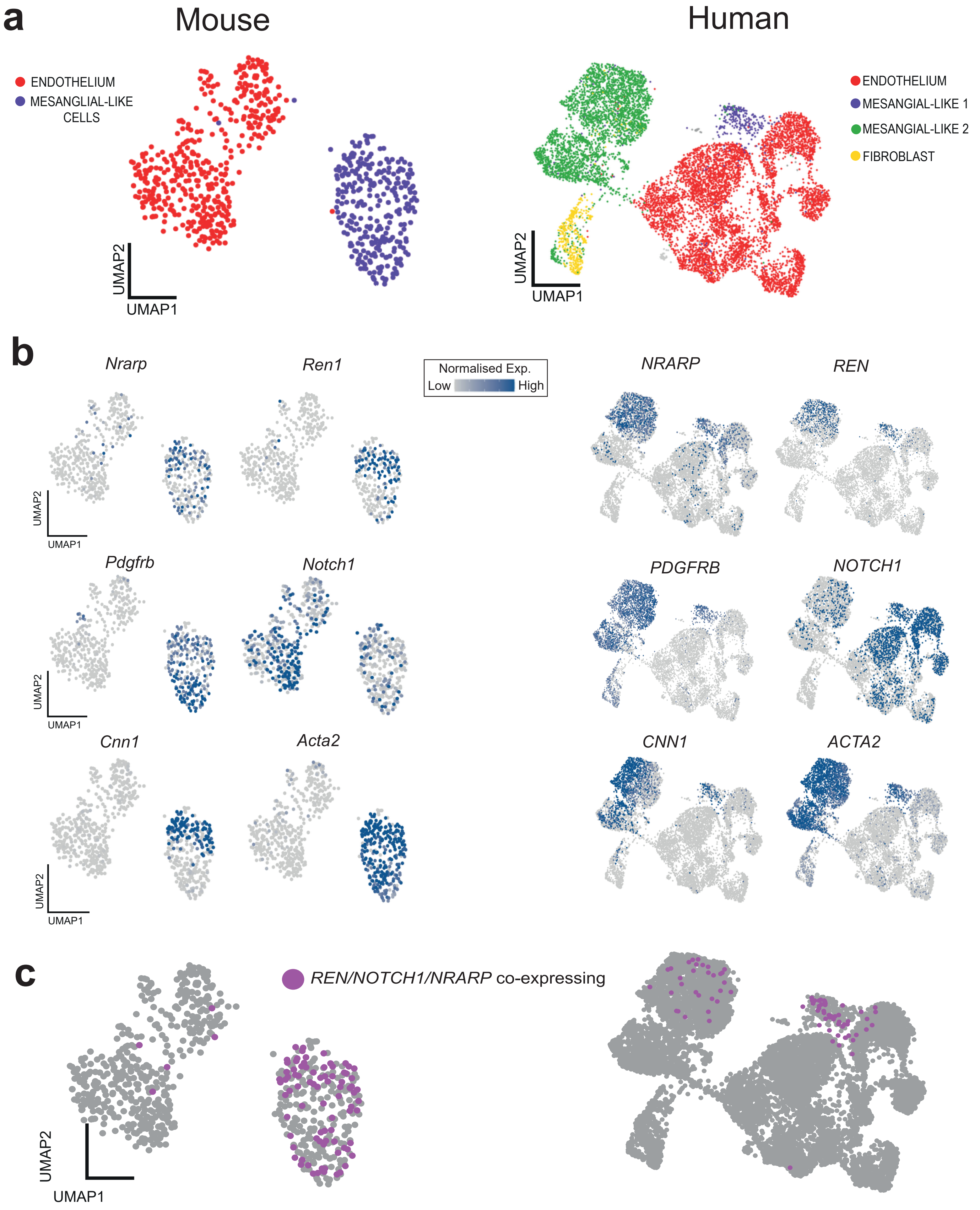

Supplementary Figure 3 - gene expression profile of mesangial like cells in healthy murine and adult human kidneys

a, Uniform Manifold Approximation and projection (UMAP) of murine and human mesangial-like cells and endothelium

b, UMAP plots showing normalised expression of mesangial-like cell marker genes, *NRARP* and *NOTCH1* across human and murine cells

c, UMAP plots showing cells co-expressing *REN*, *NOTCH1* and *NRARP* in human and murine cells

a

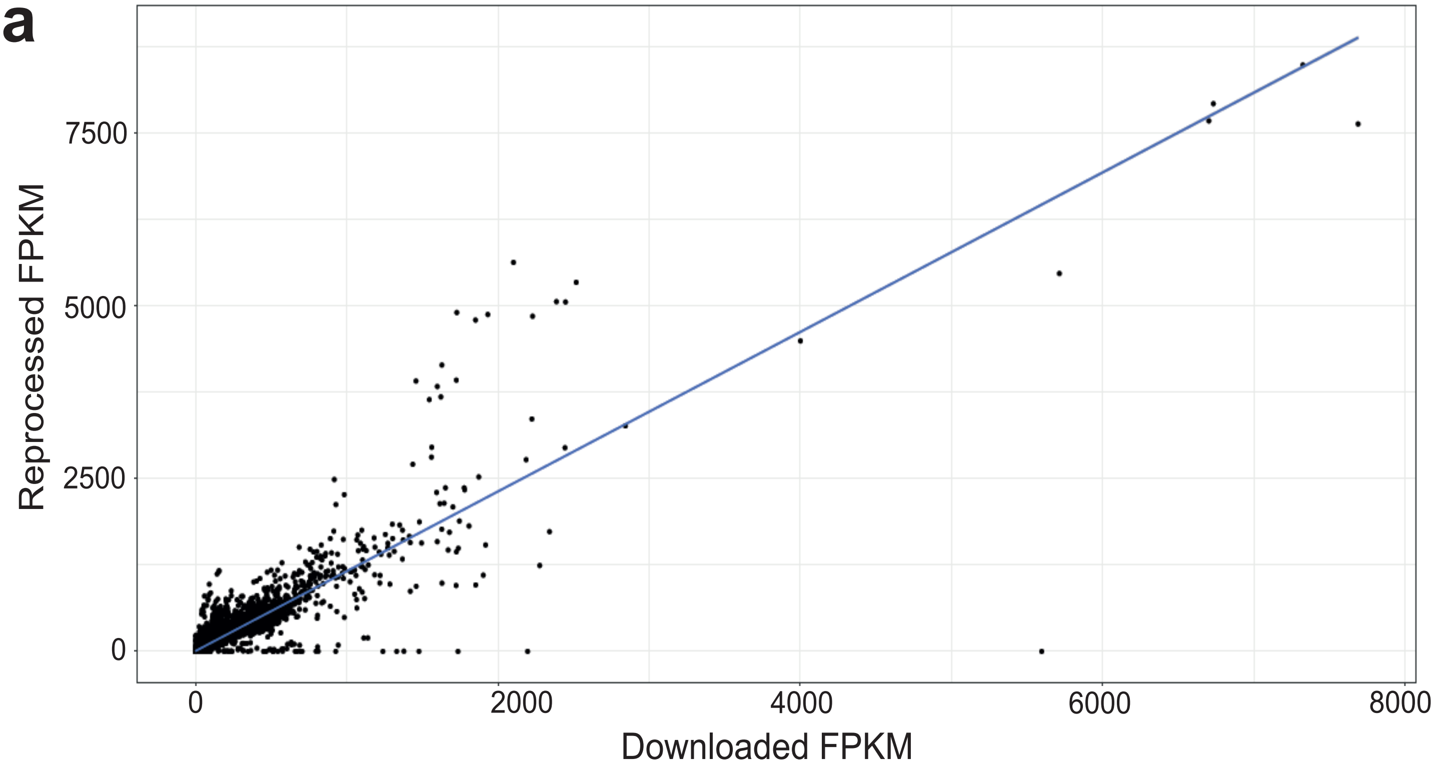

Linear Regression
Residual standard error: 76.55 on 103703 degrees of freedom
Multiple R-squared: 0.9906, Adjusted R-squared: 0.9906
F-statistic: 1.094e+07 on 1 and 103703 DF, p-value: < 2.2e-16

Supplementary Figure 4 - Expression correlation plot
a, Expression correlation plot illustrating Fragments Per Kilobase of transcript per Million mapped reads (FPKM) values for downloaded data vs. reprocessed downloaded data, values under 30,000 FKPM are shown
