# Supplementary material for: Targetable NOTCH1 rearrangements in reninoma
Source: Nat Commun. 2023 Sep 25;14:5826. doi: 10.1038/s41467-023-41118-8 (PMC10519988; doi:10.1038/s41467-023-41118-8)
Supplement: Supplementary file 3 — Description of Additional Supplementary Files [file 41467_2023_41118_MOESM3_ESM.pdf]

Description of Additional Supplementary File

Supplementary Data 1. Overview of study cohort.

Supplementary Data 2. Somatic substitutions and indels.

Supplementary Data 3. Somatic rearrangements.

Supplementary Data 4. Antibodies used for immunostaining.

Supplementary Data 5. Healthy kidney markers.

Supplementary Data 6. PD50642a markers.

Supplementary Data 7. PD54845a markers.

Supplementary Data 8. Differentially expressed genes
