# Supplementary material for: Targetable NOTCH1 rearrangements in reninoma
Source: Nat Commun. 2023 Sep 25;14:5826. doi: 10.1038/s41467-023-41118-8 (PMC10519988; doi:10.1038/s41467-023-41118-8)
Supplement: Supplementary file 5 — Reporting Summary [file 41467_2023_41118_MOESM5_ESM.pdf]

nature portfolio

Corresponding author(s): Sam Behjati
Last updated by author(s): Jul 8, 2023

Reporting Summary

Nature Portfolio wishes to improve the reproducibility of the work that we publish. This form provides structure for consistency and transparency in reporting. For further information on Nature Portfolio policies, see our Editorial Policies and the Editorial Policy Checklist.

Statistics

For all statistical analyses, confirm that the following items are present in the figure legend, table legend, main text, or Methods section.

n/a	Confirmed	
☐	☒	The exact sample size (*n*) for each experimental group/condition, given as a discrete number and unit of measurement
☒	☐	A statement on whether measurements were taken from distinct samples or whether the same sample was measured repeatedly
☐	☒	The statistical test(s) used AND whether they are one- or two-sided<br>*Only common tests should be described solely by name; describe more complex techniques in the Methods section.*
☒	☐	A description of all covariates tested
☒	☐	A description of any assumptions or corrections, such as tests of normality and adjustment for multiple comparisons
☐	☒	A full description of the statistical parameters including central tendency (e.g. means) or other basic estimates (e.g. regression coefficient) AND variation (e.g. standard deviation) or associated estimates of uncertainty (e.g. confidence intervals)
☐	☒	For null hypothesis testing, the test statistic (e.g. F, t, r) with confidence intervals, effect sizes, degrees of freedom and P value noted<br>*Give P values as exact values whenever suitable.*
☒	☐	For Bayesian analysis, information on the choice of priors and Markov chain Monte Carlo settings
☒	☐	For hierarchical and complex designs, identification of the appropriate level for tests and full reporting of outcomes
☒	☐	Estimates of effect sizes (e.g. Cohen's *d*, Pearson's *r*), indicating how they were calculated

Our web collection on statistics for biologists contains articles on many of the points above.

Software and code

Policy information about availability of computer code

Data collection	No packages were used
Data analysis	Software:<br>Burrows-Wheeler algorithm<br>CaVEMan V 1.14.1<br>Pindel V 3.3.0<br>ASCAT V 4.5.0<br>Battenberg V 3.5.3<br>BRASS V 6.3.4<br>BWcat from cgpBigWig V 1.5.2<br>STAR V 2.7.10<br>HTSeq V 0.7.2<br>Cellranger V 3.0.2<br>AlleleIntegrator V 0.9.1<br>BCFtools V 1.9<br><br>R packages:<br>Edge R V 3.32.1<br>Limma V 3.46.0<br>Glimma V 2.0.0<br>Ggplot2 V 3.3.3

Seurat V 4.0.1
SoupX V 1.48
Seurat V 4.0.1
Scrublet V 0.2.2

For manuscripts utilizing custom algorithms or software that are central to the research but not yet described in published literature, software must be made available to editors and reviewers. We strongly encourage code deposition in a community repository (e.g. GitHub). See the Nature Portfolio guidelines for submitting code & software for further information.

Data

Policy information about availability of data

All manuscripts must include a data availability statement. This statement should provide the following information, where applicable:

- Accession codes, unique identifiers, or web links for publicly available datasets
- A description of any restrictions on data availability
- For clinical datasets or third party data, please ensure that the statement adheres to our policy

The authors declare that all data supporting the findings of this study are available within the article, Source Data file and its Supplementary Information files, or from the corresponding author.
Sequencing data have been deposited at the European Genome-Phenome Archive (http://www.ebi.ac.uk/ega/) that is hosted by the European Bioinformatics Institute (accession numbers EGAD00001010888 (WGS) [https://ega-archive.org/datasets/EGAD00001010888], EGAD00001010889 (bulk RNA) [https://ega-archive.org/datasets/EGAD00001010889], EGAD00001010887 (single nuclear RNA) [https://ega-archive.org/datasets/EGAD00001010887]. The data are available under restricted access, due to data privacy laws, access may be granted following an application to the Data Access Committee, datasharing@sanger.ac.uk
Third party data used within this study are available via the corresponding references. Re-analysed reninoma data is available GSE57401 https://www.ncbi.nlm.nih.gov/geo/query/acc.cgi?acc=GSE57401
Human and mouse mesangial data is available via EGA, EGAD00001008030 [https://ega-archive.org/datasets/EGAD00001008030] and via GEO, GSE160048 [https://www.ncbi.nlm.nih.gov/geo/query/acc.cgi?acc=GSE160048].
Bulk transcriptomes of other renal tumours GSE157256 [https://www.ncbi.nlm.nih.gov/geo/query/acc.cgi?acc=GSE157256], GSE62944[https://www.ncbi.nlm.nih.gov/geo/query/acc.cgi?acc=GSE62944], and via EGA, EGAD00001008470 [https://ega-archive.org/datasets/EGAD00001008470], EGAS00001002487 [https://ega-archive.org/studies/EGAS00001002487], EGAS00001002534 [https://ega-archive.org/studies/EGAS00001002534], EGAD00001004346 [https://ega-archive.org/studies/EGAS00001002486].

Human research participants

Policy information about studies involving human research participants and Sex and Gender in Research.

Reporting on sex and gender	The sex of the patients was recorded, but no further analysis was carried out due to the limited number of patients.
Population characteristics	NA
Recruitment	NA
Ethics oversight	Case 1 (PD50642) was enrolled in the UMBRELLA study, this was approved by the national research ethics committee (London Bridge Research Ethics Comittee 12/LO/0101). Case 2 (PD54845) was enrolled in the Characterisation of the immunological and biological markers of Renal cancer progression (West of Scotland Research Ethics Comittee16/WS/0039). All patients registered (or their legal guardian) provided signed informed consent to undertake genetic testing of their samples.

Note that full information on the approval of the study protocol must also be provided in the manuscript.

Field-specific reporting

Please select the one below that is the best fit for your research. If you are not sure, read the appropriate sections before making your selection.

☒ Life sciences ☐ Behavioural & social sciences ☐ Ecological, evolutionary & environmental sciences

For a reference copy of the document with all sections, see nature.com/documents/nr-reporting-summary-flat.pdf

Life sciences study design

All studies must disclose on these points even when the disclosure is negative.

Sample size	Sample size dictated by rarity of reninomas

Data exclusions	No data was excluded.
Replication	Attempts at replication for staining were successful.
Randomization	Randomization was not applicable due to sample size.
Blinding	Blinding was not applicable due to sample size.

Reporting for specific materials, systems and methods

We require information from authors about some types of materials, experimental systems and methods used in many studies. Here, indicate whether each material, system or method listed is relevant to your study. If you are not sure if a list item applies to your research, read the appropriate section before selecting a response.

Materials & experimental systems

n/a	Involved in the study
☐	☒ Antibodies
☒	☐ Eukaryotic cell lines
☒	☐ Palaeontology and archaeology
☒	☐ Animals and other organisms
☒	☐ Clinical data
☒	☐ Dual use research of concern

Methods

n/a	Involved in the study
☒	☐ ChIP-seq
☒	☐ Flow cytometry
☒	☐ MRI-based neuroimaging

Antibodies

Antibodies used

Primary antibodies
Protein (clone); Species and Clonality; Company; Reference; Dilution
NOTCH1 (EP1238Y) rabbit monoclonal Abcam ab52627 1/150,1/300
NOTCH1 (D1E11) rabbit monoclonal Cell signalling 3608 1/200
NICD1 (Val1744; D3B8) rabbit monoclonal Cell signalling 4147 1/200
RENIN (7D3-E3) mouse monoclonal Abcam ab134783 1/200
RENIN sheep polyclonal ThermoFisher PA5-47577 1/200

Secondary antibodies
Host/Fluorophore; Target; Company; Reference; Dilution
Donkey Alexa-555 anti-rabbit Invitrogen A31572 1/500
Donkey Alexa-488 anti-mouse Invitrogen A21202 1/500
Donkey Alexa-488 anti-sheep Invitrogen A11015 1/500

Validation

Please see supplementary table 4 for more information.
Images displayed in the manuscript were acquired from assays using Abcam #ab52627 (NOTCH1 [EP1238Y]), Cell Signalling #4147 (NICD1 [Val1744; D3B8]) and Abcam #ab134783 (Renin [7D3-E3]) antibodies. Immunofluorescence assays using Cell Signaling #3608 (NOTCH1 [D1E11]) and ThermoFisher #PA5-47577 (Renin) antibodies were used for further confirmation and corroborated the results that are shown.
Anti-NOTCH1 [EP1238Y] (ab52627) rabbit monoclonal antibody was validated by the supplier for both immunohistochemistry and immunofluorescence assays in Human species and for its specificity using a NOTCH1 knockout HeLa cell line (https://www.abcam.com/products/primary-antibodies/notch1-antibody-ep1238y-ab52627.html).
Anti-NOTCH1 [D1E11] (Cell Signaling #3608) rabbit monoclonal antibody is highly cited and produced by immunization using a human NOTCH1 peptide at the C-terminus of the protein (https://www.cellsignal.com/products/primary-antibodies/notch1-d1e11-xp-rabbit-mab/3608). It was validated for immunofluorescence/ immunohistochemistry in a previous study using a knockout Notch1 mouse model and by DNA sequencing in Human tissues (Abby, Emilie et al. “Notch1 mutations drive clonal expansion in normal esophageal epithelium but impair tumor growth.” Nature genetics vol. 55,2 (2023): 232-245. doi:10.1038/s41588-022-01280-z).
Anti-NICD1 [Val1744; D3B8] (Cell Signaling #4147) rabbit monoclonal antibody is highly cited. It recognises NOTCH1 intracellular domain (NICD) and was used for immunofluorescence assays in both human and mouse species in multiple studies (https://www.cellsignal.co.uk/products/primary-antibodies/cleaved-notch1-val1744-d3b8-rabbit-mab/4147). It was used for immunofluorescence in both human and mouse tissues and validated using a knockout Notch1 mouse model in a previous study (Abby, Emilie et al. “Notch1 mutations drive clonal expansion in normal esophageal epithelium but impair tumor growth.” Nature genetics vol. 55,2 (2023): 232-245. doi:10.1038/s41588-022-01280-z). Immunostaining using each anti-NOTCH1 antibodies or anti-NICD1 antibody gave comparable results with a strong nuclear expression of the protein observed in most reninoma tumour cells and a weak or negative expression in the large majority of the cells in donor-matched and unmatched normal kidney samples.
Anti-Renin [7D3-E3] (ab134783) is a mouse monoclonal antibody targeting full length wild-type human Renin and suitable for immunostaining (https://www.abcam.com/products/primary-antibodies/renin-antibody-7d3-e3-ab134783.html). The antibody was successfully used in immunofluorescence assays in a published study to detect Renin expressing cells in postnatal healthy and nephrotic human kidneys (Kosovic, Ivona et al. “Connexin Signaling in the Juxtaglomerular Apparatus (JGA) of Developing, Postnatal Healthy and Nephrotic Human Kidneys.” International journal of molecular sciences vol. 21,21 8349. 6 Nov. 2020, doi:10.3390/ijms21218349). In our study, the detection of Renin overexpression in the cytoplasm of numerous cells in tumor sections but not or rarely in the normal kidney samples (both donor-matched and unmatched normal tissues) suggests that the antibody recognizes

Renin specifically. Renin/NOTCH1 co-staining showed that tumor cells overexpressed both proteins which is consistent with our single nuclei RNAseq data. Finally, we obtained identical results with a distinct anti-Renin antibody produced by sheep immunization using mouse myeloma cell line NS0-derived recombinant human Renin Leu24-Arg406 (ThermoFisher PA5-47577; https://www.thermofisher.com/antibody/product/Renin-Antibody-Polyclonal/PA5-47577).
